# Thrombospondin 2 expression is correlated with inhibition of angiogenesis and metastasis of colon cancer

**DOI:** 10.1038/sj.bjc.6690056

**Published:** 1999-01

**Authors:** T Tokunaga, M Nakamura, Y Oshika, Y Abe, Y Ozeki, Y Fukushima, H Hatanaka, S Sadahiro, H Kijima, T Tsuchida, H Yamazaki, N Tamaoki, Y Ueyama

**Affiliations:** Department of Pathology, Tokai University School of Medicine, Isehara, Kanagawa 259-1193, Japan; Department of Surgery II, National Defense Medical College, Tokorozawa, Saitama 359-8513, Japan; Department of Surgery, Tokai University School of Medicine, Isehara, Kanagawa 259-1193, Japan

**Keywords:** vascular endothelial growth factor, thrombospondin, colon cancer

## Abstract

Two subtypes of thrombospondin (TSP-1 and TSP-2) have inhibitory roles in angiogenesis in vitro, although the biological significance of these TSP isoforms has not been determined in vivo. We examined *TSP-1* and *TSP-2* gene expression by reverse transcription polymerase chain reaction (RT-PCR) analysis in 61 colon cancers. Thirty-eight of these 61 colon cancers were positive for *TSP-2* expression and showed hepatic metastasis at a significantly lower incidence than those without *TSP-2* expression (*P* = 0.02). *TSP-2* expression was significantly associated with M0 stage in these colon cancers (*P* = 0.03), whereas *TSP-1* expression showed no apparent correlation with these factors. The colon cancer patients with *TSP-2* expression showed a significantly low frequency of liver metastasis correlated with the cell-associated isoform of vascular endothelial growth factor (VEGF-189) (*P* = 0.0006). Vascularity was estimated by CD34 staining, and TSP-2(–)/VEGF-189(+) colon cancers showed significantly increased vessel counts and density in the stroma (*P* < 0.0001). TSP-2(–)/VEGF-189(+) colon cancer patients also showed significantly poorer prognosis compared with those with TSP-2(+) / VEGF-189(–) (*P* = 0.0014). These results suggest that colon cancer metastasis is critically determined by angiogenesis resulting from the balance between the angioinhibitory factor TSP-2 and angiogenic factor VEGF-189. © 1999 Cancer Research Campaign


*British Journal of Cancer* (1999) **79**(2), 354–359
© 1999 Cancer Research Campaign

### Thrombospondin 2 expression is correlated with inhibition of angiogenesis and metastasis of colon cancer

**T Tokunaga[1], M Nakamura[1], Y Oshika[1], Y Abe[1], Y Ozeki[2], Y Fukushima[1], H Hatanaka[1], S Sadahiro[3], H Kijima[1], T Tsuchida[1], H Yamazaki[1], N Tamaoki[1] and Y Ueyama[1]**

[1]Department of Pathology, Tokai University School of Medicine, Bohseidai, Isehara, Kanagawa 259-1193, Japan; [2]Department of Surgery II, National Defense Medical College, Namiki 3-2, Tokorozawa, Saitama 359-8513, Japan; [3]Department of Surgery, Tokai University School of Medicine, Bohseidai, Isehara, Kanagawa 259-1193, Japan

**Summary** Two subtypes of thrombospondin (TSP-1 and TSP-2) have inhibitory roles in angiogenesis in vitro, although the biological significance of these TSP isoforms has not been determined in vivo. We examined *TSP-1* and *TSP-2* gene expression by reverse transcription polymerase chain reaction (RT-PCR) analysis in 61 colon cancers. Thirty-eight of these 61 colon cancers were positive for *TSP-2* expression and showed hepatic metastasis at a significantly lower incidence than those without *TSP-2* expression ($P = 0.02$). *TSP-2* expression was significantly associated with M0 stage in these colon cancers ($P = 0.03$), whereas *TSP-1* expression showed no apparent correlation with these factors. The colon cancer patients with *TSP-2* expression showed a significantly low frequency of liver metastasis correlated with the cell-associated isoform of vascular endothelial growth factor (VEGF-189) ($P = 0.0006$). Vascularity was estimated by CD34 staining, and TSP-2(–)/VEGF-189(+) colon cancers showed significantly increased vessel counts and density in the stroma ($P < 0.0001$). TSP-2(–)/VEGF-189(+) colon cancer patients also showed significantly poorer prognosis compared with those with TSP-2(+) / VEGF-189(–) ($P = 0.0014$). These results suggest that colon cancer metastasis is critically determined by angiogenesis resulting from the balance between the angioinhibitory factor TSP-2 and angiogenic factor VEGF-189.

**Keywords:** vascular endothelial growth factor; thrombospondin; colon cancer

The prognosis of colon cancer is correlated with the presence or absence of distant metastasis. Cancer metastasis requires stromal angiogenesis including proliferation of endothelial cells and migration through extracellular matrix barriers. Angiogenesis depends on the local balance between various molecules that induce and inhibit neovascularization. In the microenvironment around successful tumours, this balance shifts from neutral to angiogenic conditions. Angiogenic factors secreted by tumours induce a vigorous angiogenic response overcoming potent inhibitors.

Thrombospondin (TSP) is a high-molecular-weight multifunctional glycoprotein which was first described as a product of platelets (Baenziger et al, 1972), released from the alpha granules in response to activation of thrombin (Lawler et al, 1986). TSP is not only one of the components of the extracellular matrix (ECM) in a wide variety of tissues (O’Shea et al, 1988; O’Shea et al, 1990), but also contains binding sites for a number of ECM and cell-associated molecules (Taraboletti et al, 1987; Sage et al, 1991). TSP is synthesized and secreted by various types of cells, i.e. fibroblasts (Jaffe et al, 1983), smooth muscle cells (Majack et al, 1987), monocytes and macrophages (Jaffe et al, 1985), osteoblasts (Gehron et al, 1989) and various neoplastic cells (Roberts et al, 1987; Zabrenetzky et al, 1994; Qian et al, 1996). TSP induces platelet aggregation (Legrand et al, 1992) and inhibits angiogenesis (Iruela et al, 1991; Weinstat et al, 1994). There are five subtypes of TSPs, namely TSP-1, TSP-2 (Labell et al, 1993), TSP-3 (Vos et al, 1992), TSP-4 (Lawler et al, 1993) and cartilage oligomeric matrix protein (Oldberg et al, 1992). Of the five structurally different TSPs, TSP-1 and TSP-2 show similarities in their molecular architecture and modulatory effects on angiogenesis (Bornstein et al, 1991; Bornstein et al, 1992; Laherty et al, 1992). Recently, TSP-1 has been implicated in progression in melanoma, lung cancer and breast cancer cell lines (Zabrenetzky et al, 1994). Transfection of TSP-1 cDNA into a human breast carcinoma cell line reduced primary tumour growth, metastatic potential and angiogenesis (Weinstat et al, 1994). TSP-1 expression inhibits angiogenesis in human glioblastoma cell lines (Hsu et al, 1996). TSP-2 has not been well characterized, although there have been a few reports of its cell biological behaviour and expression in breast cancer (Laherty et al, 1992; Bertin et al, 1997).

Vascular endothelial growth factor (VEGF) is an inducer of angiogenesis (Connolly et al, 1989; Takahashi et al, 1995; Warren et al, 1995). We demonstrated that expression of the cell-associated isoform VEGF-189 was significantly correlated with metastasis and prognosis in colon cancer (Tokunaga et al, 1998). TSP-1 and TSP-2 are potent antiangiogenic factors in colon cancer. In this study, we evaluated *TSP-1* and *TSP-2* expression in colon cancer as antiangiogenic factors, and discuss here the local balance of these factors in association with the angiogenic factor VEGF.

*Received 10 December 1997*
*Revised 28 April 1998*
*Accepted 6 May 1998*

*Correspondence to:* M Nakamura

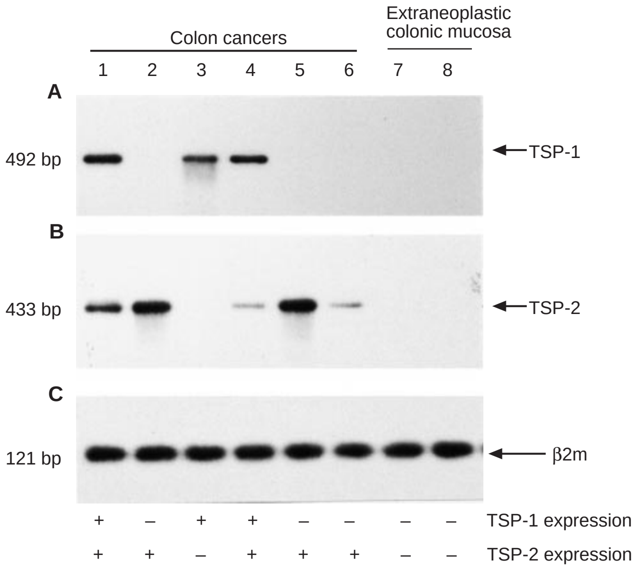

**Figure 1** Examples of *TSP-1* and *TSP-2* gene expression in colon cancers and extraneoplastic specimens: *lanes 1–6, colon cancer specimens; lanes 7 and 8, extraneoplastic colonic mucosal specimens.* (**A**) *TSP-1* expression was detected by RT-PCR with primers Th1-S and Th1-A, showing a 492-bp specific fragment. (**B**) *TSP-2* expression was detected by RT-PCR with primers Th2-S and Th2-A, showing a 433-bp specific fragment. (**C**) β2m gene expression was evaluated to qualify RNA samples

### MATERIALS AND METHODS

### Subjects and tissue samples

The subjects in this study were 61 patients (27 women and 34 men, mean age 61.4) with colon cancer (58 adenocarcinoma, three mucinous carcinoma) who underwent surgical resection between October 1989 and October 1991 at Tokai University Hospital, Kanagawa, Japan. All patients were evaluated by TNM score. Surgical specimens were rapidly frozen and stored at –80°C until analyses.

### Expression of *TSP-1* and *TSP-2* genes

We evaluated TSP mRNA expression by reverse transcription polymerase chain reaction (RT-PCR) using the following primers: for TSP-1, Th1-S, 5′-ACCGCATTCCAGAGTCTGGC-′3; Th1-A, 5′-ATGGGGACGTCCAACTCAGC-′3; and for TSP-2, Th2-S, 5′-CTGTGTCAACACTCAGCCTGGC-′3; Th2-A, 5′-TCCTTCT-CATCGGTCACACCG-′3. Reverse transcription was performed at 42°C for 60 min (1 μg total cellular RNA; 100 pM random primers, 40 U reverse transcriptase, Gibco-BRL). DNA fragments were amplified by 30 cycles of denaturation at 94°C for 1 min, annealing at 60°C for 1 min, extension at 72°C for 2 min using a Gene Amp PCR System 9600 (Perkin Elmer) and *Taq* DNA polymerase (1.3 U Toyobo, Japan). Blots (Zeta-Probe, Bio-Rad) were hybridized with photochemically labelled TSP-1- or TSP-2-specific cDNA probe (ECL; Amersham) and exposed to Kodak AR film. *TSP* gene expression was also estimated by Northern blotting analysis with total cellular RNA (15 μg, GeneScreen Plus, New England Nuclear). The quality of the RNA was estimated by RT-PCR for β2-microglobulin.

**Table 1** Univariate analysis of the associations between *TSP* gene expression and tumour characteristics

| | TSP-1 | | *P*-value | TSP-2 | | *P*-value |
|---|---|---|---|---|---|---|
| | + | – | | + | – | |
| v-factor | | | 0.498 | | | 0.711 |
| v1+ ≥ | 14 | 17 | | 20 | 11 | |
| v2+ ≤ | 11 | 19 | | 18 | 12 | |
| ly-factor | | | 1 | | | 0.739 |
| ly1+≥ | 20 | 29 | | 31 | 18 | |
| ly2+≤ | 5 | 7 | | 7 | 5 | |
| T-staging | | | 0.471 | | | 0.739 |
| T0–T2 | 6 | 6 | | 7 | 5 | |
| T3, T4 | 19 | 30 | | 31 | 18 | |
| N-staging | | | 0.131 | | | 0.561 |
| N0 | 16 | 16 | | 21 | 11 | |
| N–N3 | 9 | 20 | | 17 | 12 | |
| M-staging | | | 0.554 | | | 0.03[a] |
| M0 | 19 | 25 | | 31 | 13 | |
| M1 | 6 | 11 | | 7 | 10 | |
| Liver metastasis | | | 0.344 | | | 0.016[a] |
| Yes | 5 | 11 | | 6 | 10 | |
| No | 20 | 25 | | 32 | 13 | |
| K-*ras* mutation | | | 0.271 | | | 0.242 |
| Yes | 9 | 18 | | 19 | 8 | |
| No | 16 | 18 | | 19 | 15 | |

[a]TSP-2 expression was inversely correlated with M0 stage and liver metastasis of colon cancer ($P < 0.05$, $\chi^2$ test). The degree of venous invasion (v-factor) was classified into four groups as follows: v0, no venous invasion; v1+, minimal venous invasion, i.e. one or two foci of venous invasion in the histological sections; v2+, moderate venous invasion, i.e. three or four foci of venous invasion; and v3+, severe venous invasion, i.e. more than five invasion foci. Also, the degree of lymphatic invasion (ly-factor); ly1+, mild lymphatic invasion; ly2+, moderate lymphatic invasion; and ly3+, severe lymphatic invasion.

### Vascularization in colon cancer

Formalin-fixed, paraffin-embedded sections of the tumour tissue were examined immunohistochemically with mouse anti-human CD34 monoclonal antibody (NCL-end, Novo Castra). After blockage of endogenous peroxidase activity (methylalcohol, 3% $H_2O_2$) and non-specific binding (10% normal goat serum), specimens were incubated with anti-CD34 antibody (1:20) at room temperature for 60 min. Sections were serially incubated with biotin-labelled anti-mouse IgG (Nichirei, Tokyo, Japan) and horseradish peroxidase – conjugated streptavidin (Nichirei, Tokyo, Japan). Reaction products were visualized with 3, 3′-diamino-benzidine. Light microscopy was used to identify two regions within or immediately adjacent to the cancer containing the highest numbers of vessels. The microvessel counts and densities were evaluated at × 200 magnification (× 20 objective and × 10 ocular, 0.739 $mm^2$ per field) using a computerized image analyser (Interactive Build Analysis System, Zeiss).

### Statistical analysis

Differences in survival between subgroups of patients were compared with the log-rank test, and survival curves were plotted according to the method of Kaplan and Meier. The $\chi^2$ test or Fisher’s exact test was applied for comparisons between group

© Cancer Research Campaign 1999

**Table 2** *TSP* gene expression and VEGF mRNA isoform patterns

| ***TSP* expression** | **VEGF type 1/2[a]** | **VEGF type 3[a]** |
|---|---|---|
| TSP-1 (+) | 10 | 15 |
| TSP-1 (–) | 19 | 17 |
| TSP-2 (+) | 18 | 20 |
| TSP-2 (–) | 11 | 12 |

[a]Types 1/2 expressed VEGF-121 and/or VEGF-165. Type 3 expressed VEGF-121, –165 and –189.

**Table 3** Associations between *TSP-2* gene expression linked with VEGF mRNA isoform patterns and tumour characteristics

| | **VEGF type 1/2 TSP-2 (+)** | **VEGF type 3 TSP-2 (–)** | ***P*-value** |
|---|---|---|---|
| v-factor | | | 0.0236[a] |
| v1+ ≥ | 13 | 3 | |
| v2+ ≤ | 5 | 9 | |
| ly-factor | | | 0.4181 |
| ly1+ ≥ | 14 | 7 | |
| ly2+ ≤ | 4 | 5 | |
| Vessel counts | 37 ± 16 | 77 ± 23 | < 0.0001[b] |
| Vessel density | 2.9 ± 1.2 | 6.8 ± 2.0 | < 0.0001[b] |
| T-staging | | | 0.6599 |
| T0–T2 | 3 | 3 | |
| T3, T4 | 15 | 9 | |
| N-staging | | | 0.3915 |
| N0, N1 | 15 | 8 | |
| N2, N3 | 3 | 4 | |
| M-staging | | | 0.0012[c] |
| M0 | 16 | 3 | |
| M1 | 2 | 9 | |
| Liver metastasis | | | 0.0006[c] |
| Yes | 1 | 8 | |
| No | 17 | 4 | |

Significance of differences was evaluated by $\chi^2$ test[a], ANOVA[b], Fisher's test[c].

frequencies. The statistical significance of differences in mean vessel counts and density among the groups were examined by ANOVA. Multiple comparisons were performed by the Bonferroni method.

### RESULTS

### *TSP-1* and *TSP-2* gene expression

Four of 61 colon cancer specimens expressed *TSP-1* but not *TSP-2*, whereas 17 expressed *TSP-2* but not *TSP-1* as shown by RT-PCR analysis (examples are shown in Figure 1A and B). Twenty-one of the 61 colon cancers coexpressed both *TSP-1* and *TSP-2* genes. The extraneoplastic colonic mucosal specimens demonstrated little *TSP* gene expression. *TSP-1* expression was detected in 7 out of 36 extraneoplastic colonic mucosal specimens by RT-PCR, although *TSP-1* gene expression was not detectable in the extraneoplastic tissue by Northern blotting. Moreover, *TSP-2* gene expression of colon cancer specimens (38 out of 61) was found at a significantly higher incidence than in extraneoplastic tissue (3 out of 36). Northern blotting analyses confirmed faint

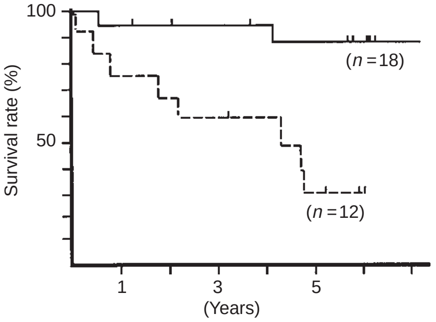

**Figure 2** The prognosis of colon cancer patients is shown on a Kaplan–Meier plot (generalized log-rank test). The prognosis of patients with expression of *TSP-2* and type 1 or 2 VEGF isoform pattern [TSP-2(+)/VEGF-189(–), solid line] was significantly better than that of patients with type 3 VEGF isoform pattern and lacking *TSP-2* expression [TSP-2(–)/VEGF-189(+), broken line] ($P = 0.0014$)

*TSP-2* gene expression in 12 out of 38 colon cancer specimens with definite *TSP-2* expression by RT-PCR, whereas the levels of expression were varied (data not shown). Southern blotting analyses showed neither amplification nor rearrangement of the *TSP-2* gene (data not shown).

### Correlation between *TSP* expression and distant metastasis

The patients with colon cancers showing *TSP-2* expression had a significantly lower incidence (6 out of 38, 15.8%) of liver metastasis than those (10 out of 23, 43.5%) without TSP-2 expression ($P = 0.02$, $\chi^2$ test, Table 1). Six out of the 16 (37.5%) patients with hepatic metastatic lesions showed *TSP-2* expression, whereas 32 out of the 45 patients (71.1%) without liver metastasis showed *TSP-2* expression. *TSP-2* expression was inversely correlated with the distant metastasis (M1 stage) of colon cancer ($P = 0.03$, $\chi^2$-test, Table 1). *TSP-2* expression was not correlated with tumour size (Tx stage) or nodal involvement (Nx stage), and expression of *TSP-1* was not correlated with any of the clinical characteristics examined (Table 1).

### Association between *TSP* gene expression and VEGF isoform

Previously, we reported that VEGF mRNA isoform expression patterns in colon cancer could be divided into three types: type 1, VEGF-121; type 2, VEGF-121/165; and type 3, VEGF-121/165/189 (Tokunaga et al, 1998). Three out of the 61 colon cancers showed type 1 expression. Twenty-six out of the 61 patients showed type 2 expression, and the remaining 32 showed the type 3 pattern. None of the tumours showed VEGF-206 expression. Twelve out of the 16 (75.0%) patients with hepatic metastatic lesions showed the type 3 pattern, whereas 20 out of the 45 patients (44.4%) without liver metastasis showed this isoform expression pattern. We analysed the correlation between *TSP* gene expression and VEGF mRNA isoform pattern (Table 2). Ten out of 25 colon cancers expressing *TSP-1* showed types 1 and 2 VEGF isoform patterns. Fifteen of those expressing *TSP-1* showed type 3 VEGF isoform pattern. Eighteen out of 38 colon cancers expressing the *TSP-2* gene showed types 1 and 2 VEGF isoform patterns, whereas 20 of those expressing *TSP-2* showed the type 3

 *© Cancer Research Campaign 1999*

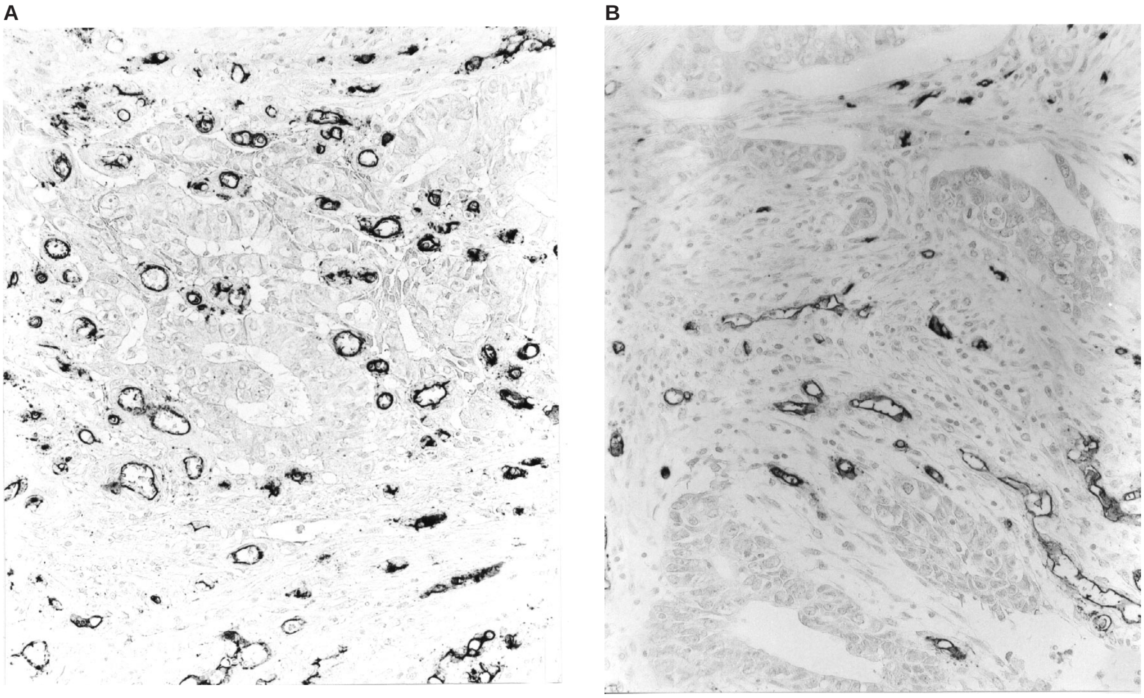

**Figure 3** Vascularization in colon cancer was demonstrated by immunostaining for CD34. (**A**) Colon cancer [TSP-2(–)/VEGF-189(+)] showed significantly increased vascular density. (**B**) Colon cancer [TSP-2(+)/VEGF-189(–)] showed moderate vascular density (× 200)

pattern. The type 3 VEGF isoform pattern was observed in 12 colon cancers without *TSP-2* gene expression. There was no significant correlation between *TSP* gene expression and VEGF isoform expression pattern (Table 2). Patients with colon cancer expressing *TSP-2* mRNA without VEGF-189 expression showed hepatic metastasis at a significantly lower incidence [TSP-2(+)/V189(–) group; 1 out of 18, 5.6%] than those with colon cancer expressing no TSP-2 with VEGF-189 expression [TSP-2(–)/V189(+) group; 8 out of 12, 75.0%] ($P = 0.0006$, Fisher's test, Table 3). TSP-2(+)/V189(–) group patients showed significantly better prognosis ($P = 0.0014$, log rank test, Figure 2) than the TSP-2(–)/V189(+) group.

### Vascularization and *TSP* expression

The mean vessel count and density in all colon cancers examined were 58.4 ± 27.7 per × 200 fields (range 16–153) and 4.7% ± 2.8% per × 200 fields (range 1.1–12.1) respectively. The mean vessel count in TSP-2(+)/V189(–) group was 36.5 ± 15.9 (range 16–70). That of the TSP-2(–)/V189(+) group was 77.1 ± 22.6 per × 200 fields (range 45–146). However, those of TSP-2(+)/V189(+) and TSP-2(–)/V189(–)group were 62.5 ± 38.7 and 52.5 ± 41.4 respectively. The mean vessel density in TSP-2(+)/V189(–) group was 2.9% ± 1.2% per × 200 fields (range 1.1–5.2), whereas that in the TSP-2(–)/V189(+) group was 6.8% ± 2.0% per × 200 fields (range 2.9–12.1). Those of TSP-2(+)/V189(+) and TSP-2(–)/V189(–) groups were 5.0 ± 4.3 and 4.5 ± 3.6 respectively. Significant differences were found among the four groups in vessel counts ($P < 0.0001$, ANOVA test) and vessel density ($P < 0.0001$, ANOVA test). The most significant difference was found between the TSP-2(+)/V189(–) and TSP-2(–)/V189(+) groups ($P < 0.0001$, Bonferroni test) (Figure 3, Table 3). Patients with colon cancer expressing VEGF-189 without *TSP-2* expression showed involvement of veins at a significantly higher incidence ($P = 0.0236$, Fisher's test, Table 3). There were no correlations between *TSP-1* or *TSP-2* mRNA expression and any histological feature of colon cancer examined (Table 1).

### DISCUSSION

Angiogenesis in the stroma is a major factor contributing to distant metastasis of colon cancers. Stromal angiogenesis control is based on the balance between various angiogenic cytokines and angioinhibitory factors. Thrombospondin (TSP) is an antiangiogenic factor (Iruela et al, 1991; Weinstat et al, 1994; Zabrenetzky et al, 1994; Volpert et al, 1995; Hsu et al, 1996). We detected TSP transcripts in 69% of primary human colon cancer specimens. It is possible that variable amounts of stromal RNA were copurified from the tumours in these bulk studies. *TSP-1* expression was detected in seven specimens of 36 extraneoplastic colonic mucosa by RT-PCR, whereas none of these were detected by Northern blotting. *TSP-2* expression was detected in 3 out of 36 extraneoplastic colonic mucosal specimens by RT-PCR. This was a significantly lower frequency than that in cancer specimens. Although it is still unclear whether the neoplastic cells or stromal cells predominantly express *TSP-1* or *TSP-2*, cancer tissue specimens showed expression of these genes at increased frequency and intensity. It was reported that *TSP* expression was more intense in the stromal tissues within and immediately adjacent to tumours (Clezardin et al, 1993; Grossfeld et al, 1996), whereas TSP was localized to some normal tissues including peritubular connective tissue of the kidney, the basement membrane regions beneath glandular epithelium in the lung, epidermal–dermal junctions, and the interstitium in skeletal muscles (Wight et al, 1985). The results presented in this study supported the view that TSP is more predominantly expressed in colon cancer than in the extraneoplastic tissue.

© Cancer Research Campaign 1999

Five different subtypes of human TSP have been identified. Among the TSP subtypes, TSP-2 showed considerable protein sequence similarity to that of TSP-1 which is known to be functionally critical. TSP-1 and TSP-2 are clearly distinct gene products (Labell et al, 1993), although both modulate angiogenesis (Volpert et al, 1995). TSP-2 blocks the migration of capillary endothelial cells evoked by a variety of inducers, and inhibits neovascularization in the rat cornea (Panetti et al, 1997). Recombinant murine TSP-2 inhibited bovine aortic endothelial cell proliferation at doses similar to those at which TSP-1 also showed this effect (Volpert et al, 1995). In various neoplastic cell lines, the level of *TSP-1* expression showed an inverse correlation with malignant progression (Weinstat et al, 1994; Zabrenetzky et al, 1994; Hsu et al, 1996). Recently, tumour progression and angiogenesis in bladder cancer were reported to be associated with *TSP-1* expression (Grossfeld et al, 1997). It has been postulated that TSP-1 modulates tumour progression through its inhibitory effect on tumour angiogenesis. However, there have been few reports concerning *TSP-2* expression encoded by genes other than *TSP-1* and functions in neoplasms including colon cancer. Two specific primers were used to confirm *TSP-1* or *TSP-2* gene expression accurately. Putative misamplified RT-PCR products do not show confusing cDNA fragment lengths (within 200 bp difference) compared with the TSP-1 or TSP-2-specific fragments, even if the primers misreact because of the 65% sequence homology. Moreover, we also confirmed that TSP-1/TSP-2-specific probes did not crossreact with each other under the conditions used for Southern blotting. The RT-PCR procedures used in this study were sufficient to specifically detect TSP-1 and TSP-2 mRNA. The results of our study demonstrated that TSP-2 was more predominantly expressed in colon cancer than TSP-1. We showed that the *TSP-2* expression was inversely and significantly correlated with liver metastasis and M1 stage in colon cancer, whereas *TSP-1* expression did not show any such correlations. These results suggested that TSP-2 is a major inhibitory factor against distant metastasis of colon cancer.

*TSP-2* expression exhibited a significant correlation with stromal vessel counts and density when they were analysed with expression of the cell-associated isoform VEGF-189. Those colon cancers expressing VEGF-189 without TSP-2 [TSP-2(–)/VEGF-189(+)] showed almost double the number and density of vessels compared with TSP-2(+)/VEGF-189(–) colon cancers. The prognostic significance of neovascularization in solid neoplasms has been demonstrated in malignant melanoma (Graham et al, 1994), breast (Weidner et al, 1993), prostate (Weidner et al, 1991), bladder (Grossfeld et al, 1997), stomach (Maeda et al, 1995) and non-small-cell lung cancer (Macchiarini et al, 1992). Vessel counts were significantly correlated with time to recurrence in node-negative colon cancer (Takahashi et al, 1997). This study showed that stromal angiogenesis is finely controlled by TSP-2 as well as VEGF-189 expression, and that *TSP-2* expression is of prognostic significance in colon cancer. Angiogenic phenotype which may be able to support tumorigenicity can arise in a stepwise fashion in response to both a decrease in the secretion of inhibitors and the sequential up-regulation of the secretion of inducers of angiogenesis (Volpert et al, 1997). The close correlation and local balance between tissue stable TSP-2 and cell-associated VEGF-189 shown in this study are critical for the progression of colon cancer. *TSP-1* expression showed no significant correlations with angiogenesis in colon cancer.

Oncogenes influence angiogenesis by encoding secreted proteins that are themselves angiogenic factors or by stimulating cells to secrete angiogenic factors as well as enzymes that enhance angiogenesis (Stellmach et al, 1996). There was no apparent correlation between activated *ras* oncogene and *TSP-2* expression, whereas the K-*ras* oncogene is known to stimulate secretion of VEGF (Rak et al, 1995). We also detected *TSP-2* expression in five out of six patients with transforming growth factor β (TGF-β) expression, although TGF-β was reported to be associated with *TSP* gene expression (Laiho et al, 1991). It is not clear which factors affect TSP-2 mRNA expression in colon cancers.

### ACKNOWLEDGEMENTS

This work was supported in part by a Grant-in-Aid for Scientific Research from the Ministry of Education, Science and Culture (MN, 09670205; HK, 9670239; NT, 07457588; 08877040; YU, 09680835) and Tokai University School of Medicine Research Aid (HK, YO and TT). We thank Mr Yuichi Tada, Mr Masashi Tomizawa, Mr Johbu Itoh and Ms Kyoko Murata for technical assistance.

### REFERENCES

Baenziger NL, Brodie GN and Majerus PW (1972) Isolation and properties of a thrombin sensitive protein of human platelets. *J Biol Chem* **247**: 2723–2731

Bertin N, Clezardin P, Kubiak R and Frappart L (1997) Thrombospondin-1 and -2 messenger RNA expression in normal, benign, and neoplastic human breast tissues: correlation with prognostic factors, tumor angiogenesis, and fibroblastic desmoplasia. *Cancer Res* **57**: 396–399

Bornstein P (1992) Thrombospondins: structure and regulation of expression. *FASEB J* **6**: 3290–3299

Bornstein P, O'Rourke K, Wikstrom K, Wolf FW, Katz R, Li P and Dixit VM (1991) A second, expressed thrombospondin gene (Thbs2) exists in the mouse genome. *J Biol Chem* **266**: 12821–12824

Clezardin P, Frappart L, Clerget M, Pechoux C and Delmas PD (1993) Expression of thrombospondin (TSP1) and its receptors (CD36 and CD51) in normal, hyperplastic, and neoplastic human breast. *Cancer Res* **53**: 1421–1430

Connolly DT, Heuvelman DM, Nelson R, Olander JV, Eppley BL, Delfino JJ, Siegel NR, Leimgruber RM and Feder J (1989) Tumor vascular permeability factor stimulates endothelial cell growth and angiogenesis. *J Clin Invest* **84**: 1470–1478

Gehron RP, Young MF, Fisher LW and McClain TD (1989) Thrombospondin is an osteoblast-derived component of mineralized extracellular matrix. *J Cell Biol* **108**: 719–727

Graham C, Rivers J, Kerbel R, Stankiewicz K and White W (1994) Extent of vascularization as a prognostic indicator in thin (< 0.76 mm) malignant melanomas. *Am J Pathol* **145**: 510–514

Grossfeld GD, Shi SR, Ginsberg DA, Rich KA, Skinner DG, Taylor CR and Cote RJ (1996) Immunohistochemical detection of thrombospondin-1 in formalin-fixed, paraffin-embedded tissue. *J Histochem Cytochem* **44**: 761–766

Grossfeld GD, Ginsberg DA, Stein JP, Bochner BH, Esrig D, Groshen S, Dunn M, Nichols PW, Taylor CR, Skinner DG and Cote RJ (1997) Thrombospondin-1 expression in bladder cancer: association with p53 alterations, tumor angiogenesis, and tumor progression. *J Natl Cancer Inst* **89**: 219–227

Hsu SC, Volpert OV, Steck PA, Mikkelsen T, Poverini PJ, Rao S, Chou P and Bouck N (1996) Inhibition of angiogenesis in human glioblastomas by chromosome 10 induction of thrombospondin-1. *Cancer Res* **56**: 5684–5691

Iruela-Arispe ML, Bornstein P and Sage H (1991) Thrombospondin exerts an anti-angiogenic effect on cord formation by endothelial cells in vitro. *Proc Natl Acad Sci USA* **88**: 5026–5030

Jaffe EA, Ruggiero JT, Leung LLK, Doyle MJ, McKeon-Longo PJ and Mosher DF (1983) Cultured human fibloblasts synthesize and secrete thrombospondin and incorporate it into extracellular matrix. *Proc Natl Acad Sci USA* **80**: 998–1002

Jaffe EA, Ruggiero JT and Falcon DJ (1985) Monocytes and macrophages synthesize and secrete thrombospondin. *Blood* **65**: 79–84

Labell TL and Byers PH (1993) Sequence and characterization of the complete human thrombospondin-2 cDNA: potential regulatory role for the 3′ untranslated region. *Genomics* **17**: 225–229

Laherty CD, O'Rourke K, Wolf FW, Katz R, Seldin MF and Dixit VM (1992) Characterization of mouse thrombospondin 2 sequence and expression during cell growth and development. *J Biol Chem* **267**: 3274–3281

 © Cancer Research Campaign 1999

Laiho M, Ronnstrand L, Heino J, Decaprio JA, Ludlow JW, Livingston DM and Massague J (1991) Control of junB and extracellular matrix protein expression by transforming growth factor-beta 1 is independent of simian virus 40T antigen-sensitive growth-sensitive growth-inhibitory events. *Mol Cell Biol* **11**: 972–978

Lawler J (1986) The structural and functional properties of thrombospondin. *Blood* **67**: 112–123

Lawler J, Duquette M, Whittaker CA, Adams JC, McHenry K and Desimone DW (1993) Identification and characterization of thrombospondin-4, a new member of the thrombospondin gene family. *J Cell Biol* **120**: 1059–1067

Legrand C, Thibert V, Dubernard V, Begault B and Lawler J (1992) Molecular requirements for the interaction of thrombospondin with thrombin-activated human platelets: modulation of platelet aggregation. *Blood* **79**: 1995–2003

Macchiarini P, Fontanini G, Hardin MJ, Squartini F and Angeletti CA (1992) Relation of neovascularization to metastasis of non-small-cell lung cancer. *Lancet* **340**: 145–146

Maeda K, Chung YS, Takatsuka S, Ogawa Y, Sawada T, Yamashita Y, Onoda N, Kato Y, Nitta A, Arimoto Y, Kondo M and Sawa M (1995) Tumor angiogenesis as a predictor of recurrence in gastric carcinoma. *J Clin Oncol* **13**: 477–481

Majack RA, Mildbrandt J and Dixit VM (1987) Induction of thrombospondin messenger RNA levels occurs as an immediate primary response to platelet-derived growth factor. *J Biol Chem* **262**: 8821–8825

Oldberg A, Antonsson P, Lindblom K and Heinegard D (1992) COMP (cartilage oligomeric matrix protein) is structurally related to the thrombospondins. *J Biol Chem* **267**: 22346–22350

O'Shea KS and Dixit VM (1988) Unique distribution of the extracellular matrix component thrombospondin in the developing mouse embryo. *J Cell Biol* **107**: 2737–2748

O'Shea KS, Liu LHJ, Kinnunen LH and Dixit VM (1990) Role of the extracellular matrix protein thrombospondin in the early development of the mouse embryo. *J Cell Biol* **111**: 2713–2723

Panetti TS, Chen H, Misenheimer TM, Getzler SB and Mosher DF (1997) Endothelial cell mitogenesis induced by LPA: inhibition by thrombospondin-1 and thrombospondin-2. *J Lab Clin Med* **129**: 208–216

Qian X and Tuszynski GP (1996) Expression of thrombospondin-1 in cancer: a role in tumor progression. *Proc Soc Exp Biol Med* **212**: 199–207

Rak J, Mitsuhashi Y, Bayko L, Filmus J, Shirasaws S, Sasazuki T and Kerbel RS (1995) Mutant *ras* oncogenes upregulate VEGF/VPF expression: implications for induction and inhibition of tumor angiogenesis. *Cancer Res* **55**: 4575–4580

Roberts DD, Sherwood JA and Ginsburg V (1987) Platelet thrombospondin mediates attachment and spreading of human melanoma cells. *J Cell Biol* **104**:131–139

Sage EH and Bornstein P (1991) Extracellular proteins that modulate cell-matrix interactions. *J Biol Chem* **266**: 14831–14834

Stellmach V, Volpert OV, Crawford SE, Lawler J, Hynes RO and Bouck N (1996) Tumour suppresser genes and angiogenesis. *Eur J Cancer* **32A**: 2394–2400

Takahashi Y, Kitadai Y, Bucana CD, Cleary RK and Ellis LM (1995) Expression of vascular endothelial growth factor and its receptor, KDR, correlates with vascularity, metastasis, and proliferation of human colon cancer. *Cancer Res* **55**: 3964–3968

Takahashi Y, Tucker SL, Kitadai Y, Koura AN, Bucana CD, Cleary KR and Ellis LM (1997) Vessel counts and expression of vascular endothelial growth factor as prognostic factors in node-negative colon cancer. *Arch Surg* **132**: 541–546

Taraboletti G, Roberts DD and Liotta LA (1987) Thrombospondin-induced tumor cell migration: haptotaxis and chemotaxis are mediated by different molecular domains. *J Cell Biol* **105**: 2409–2415

Tokunaga T, Oshika Y, Abe Y, Ozeki Y, Sadahiro S, Kijima H, Tsuchida T, Yamazaki H, Ueyama Y, Tamaoki N and Nakamura M (1998) Vascular endothelial growth factor (VEGF) mRNA isoform expression pattern is correlated with liver metastasis and poor prognosis in colon cancer. *Br J Cancer* **77**: 996–1002

Volpert OV, Tolsma SS, Pellerin S, Feige JJ, Chen H, Mosher DF and Bouck N (1995) Inhibition of angiogenesis by thrombospondin-2. *Biochem Biophys Res Commun* **217**: 326–332

Volpert OV, Dameron KM and Bouck N (1997) Sequential development of an angiogenic phenotype by human fibloblasts progressing to tumorgenicity. *Oncogene* **14**: 1495–1502

Vos HL, Devarayalu S, De Vries Y and Bornstein P (1992) Thrombospondin 3 (Thbs3), a new member of the thrombospondin family. *J Biol Chem* **267**: 12192–12196

Warren SR, Yuan H, Matli RM, Gillett AN and Ferrara N (1995) Regulation by vascular endothelial growth factor of human colon cancer tumorgenesis in a mouse model of experimental liver metastasis. *J Clin Invest* **95**: 1789–1797

Weidner N, Semple JP, Welch WR, Folkman J (1991) Tumor angiogenesis and metastasis-correlation in invasive breast carcinoma. *N Engl J Med* **324**: 1–8

Weidner N, Carro PR, Flax J, Blumenfeld W and Folkman J (1993) Tumor angiogenesis correlates with metastasis in invasive prostate carcinoma. *Am J Pathol* **143**: 401–409

Weinstat-Saalow DL, Zabrenetzky VS, Vanhoutte K, Frazier WA, Roberts DD and Steeg PS (1994) Transfection of thrombospondin 1 complementary DNA into a human breast carcinoma cell line reduces primary tumor growth, metastatic potential, and angiogenesis. *Cancer Res* **54**: 6504–6511

Wight TN, Raugi GJ, Mumby SM and Bornstein P (1985) Light microscopic immunolocation of thrombospondin in human tissues. *J Histochem Cytochem* **33**: 295

Zabrenetzky V, Harris CC, Steeg PS and Roberts DD (1994) Expression of the extracellular matrix molecule thrombospondin inversely correlates with malignant progression in melanoma, lung, and breast carcinoma cell lines. *Int J Cancer* **59**: 191–195

*© Cancer Research Campaign 1999* 
